# Investment in preventive health needs to be seen as a driver of economic development

**DOI:** 10.1371/journal.pmed.1005074

**Published:** 2026-05-05

**Authors:** Francesca Colombo, Sabine Vuik, Marion Devaux, Michele Cecchini

**Affiliations:** OECD, Health Division, Directorate for Employment, Labour and Social Affairs, Paris, France

## Abstract

In this Perspective, Francesca Colombo and colleagues outline why strategic investment in preventive health is critical to reduce health system costs, boost productivity and sustain long-term prosperity.

## Teaser

Prevention delivers major gains in health and productivity but remains underfunded due to governance and technical barriers. Strategic investment in prevention is critical to reduce health system costs, boost productivity and sustain long-term prosperity.

Health and wealth have long been intertwined, but the depth of this interdependence is often overlooked in strategies for economic growth. Traditional growth models emphasize two fundamental drivers of economic prosperity: human capital and physical capital investment. Good population health is a cornerstone of both.

Human capital refers to the skills, knowledge, and productivity of the workforce. Analyses by the Organisation for Economic Co-operation and Development (OECD) demonstrate that healthier individuals are significantly more likely to be employed and, once employed, exhibit higher productivity due to reduced absenteeism and presenteeism, the phenomenon of being at work but performing below capacity. For instance, compared to healthy people, those with one or more chronic conditions are 16% less likely to be employed, and this figure raises to 30% for those with two or more conditions [1]. For instance, evidence shows that early mental health conditions reduce educational attainment and later occupational and social outcomes, while poor physical and mental health in adulthood is associated with unemployment and lower socioeconomic status [2]. These educational disadvantages then translate into lower labor market performance and earnings later in life.

Physical capital grows through investment in productive assets such as machineries and infrastructures. The level of capital investment depends on the resources available after meeting current spending needs, as only the remaining funds can be allocated to investment in productive assets. OECD countries spend on average about 9.3% of their gross domestic product on health, mostly for treating diseases [3]. If populations were healthier, part of these resources could be redirected to physical and other capital investment, enhancing uses such as innovation and education.

So, good health is good for economies, but what is the best way to achieve good health? For many countries, investing in effective prevention for chronic diseases delivers greater health and economic gains than prioritizing treatment improvements [4]. Preventive interventions, such as tobacco control, obesity reduction strategies, and vaccination programs avert costly diseases and reduce healthcare expenditure by reducing the need for people to receive treatments and care, and thereby sustain their workforce participation.

Yet, despite all the evidence, the reality tells a different story, with prevention remaining undervalued and, therefore, underfunded. Across OECD countries, spending on prevention represents only a small fraction of total health budgets, on average around 3%, making it the least funded component of health systems [3].

Several structural and behavioral factors explain why prevention struggles to secure adequate funding and recognition. One key issue is that preventive interventions are often judged against unrealistically stringent benchmarks. While treatments are considered acceptable if they meet cost-effectiveness thresholds—defining what health systems are willing to pay for health gains, such as £25,000 to £35,000 per quality-adjusted life year in the UK—prevention is often expected to be cost-saving before policymakers view it as a worthwhile investment [5]. This double standard undervalues prevention interventions that still deliver substantial long-term health benefits at a cost comparable to curative treatments.

Another challenge lies with prevention measures that target healthy individuals who may not feel a need for action, such as in the case of vaccinations or promotion of healthier lifestyles. This lack of urgency weakens political pressure and can even provoke resistance among target populations when interventions require changes to established habits or lifestyles. Moreover, interventions such as those addressing childhood obesity or promoting healthy behaviors, yield returns only decades later [6], further discouraging action. These benefits, while significant at the population level, are difficult to quantify for policymakers focused on current urgencies, making policy prioritization harder.

Finally, prevention responsibilities are spread across a wide range of actors, including health authorities as well as education and environment ministries, local governments, and many others. This broad distribution creates significant coordination challenges. The absence of standardized, consensus-based assessment models of the benefits of prevention interventions further complicates decision-making and resource allocation.

So, is prevention doomed to a secondary role? Research shows that the answer should be a resounding no. On the contrary, prevention should be recognized as a fundamental driver of sustainable economic growth thanks to its positive return on investment. Translating this vision into action is easier said than done, however, and demonstrating the evidence on the economic returns from investment in prevention is only the first step. Health authorities rather need to implement a comprehensive three-pronged strategy.

First, the co-benefits that prevention interventions deliver across other sectors of the economy should be systematically recognized and emphasized. The OECD Strategic Public Health Planning (SPHeP) framework and its related modeling approaches [7] illustrate how investments in population health generate advantages beyond health itself. For example, cancer policies focused on encouraging healthier diets can prevent 140,000 cases of cancer per year in the OECD, save EUR 12.2 billion per year in cancer health expenditure, and reduce greenhouse gas emissions by 304 Mt of CO_2_-equivalent per year (comparable to 72 million gasoline-powered cars), whilst increasing human capital and fostering economic growth [8]. This evidence empowers health authorities to build alliances with other governmental agencies and stakeholders, showing that prevention measures not only improve well-being but also help partners achieve their own strategic objectives.

Second, countries should invest in integrated governance models that keep prevention at the forefront, even during complex political contexts or periods of fiscal constraint. The Netherlands’ efforts to strengthen governance for prevention illustrate this approach [9]. This becomes even more effective when combined with robust appraisal mechanisms such as the UK’s Green Book, enabling preventive proposals to be evaluated on the same basis as other investments [10], and with comprehensive performance indicators like those used in New Zealand to monitor progress across social, economic, and environmental metrics [11].

Third, innovative financing pathways can be unlocked to bring fresh resources to prevention to supplement allocations from strained government budgets. A significant opportunity lies in mobilizing private sector investment. For instance, OECD analyses show that investing in a healthier workforce not only boosts productivity but also brings significant advantages for companies investing in prevention interventions, from lowering health-related costs and enhancing corporate image to attracting and retaining talent, improving employee engagement, and potentially raising stock values [12]. Yet private investment in prevention alone is not enough. Other mechanisms, such as taxes on unhealthy products [13], financing models that tie payments to achieving specific health outcomes (such as improving vaccination coverage), and public health bonds (where investors provide upfront funding for prevention and are repaid if targets are met), are increasingly being explored as viable options [14]. Blending these new approaches with regular budget allocations helps create sustainable funding streams while aligning incentives for better health outcomes.

Prevention reduces future healthcare costs and boosts productivity, benefits that compound over time. Investing in prevention should be recognized not as a luxury but as a strategic asset to sustain economic development, enhance resilience, and promote societal well-being.

## Abbreviations

OECD: Organisation for Economic Co-operation and Development
SPHeP: Strategic Public Health Planning
